# Comparison of double-balloon and single-balloon enteroscope for therapeutic endoscopic retrograde cholangiography after Roux-en-Y small bowel surgery

**DOI:** 10.1186/s12876-016-0512-6

**Published:** 2016-08-22

**Authors:** Michael De Koning, Tom G. Moreels

**Affiliations:** Department of Gastroenterology & Hepatology, Antwerp University Hospital, Wilrijkstraat 10, B-2650 Antwerp, Belgium

**Keywords:** ERCP, Balloon-assisted enteroscopy, Roux-en-Y

## Abstract

**Background:**

Roux-en-Y reconstructive surgery excludes the biliopancreatic system from conventional endoscopic access. Balloon-assisted enteroscopy allows therapeutic endoscopic retrograde cholangiopancreatography (ERCP) in these patients, avoiding rescue surgery.

The objective of the current study is to compare success and complication rate of double-balloon (DBE) and single-balloon enteroscope (SBE) to perform ERCP in Roux-en-Y patients.

**Methods:**

Seventy three Roux-en-Y patients with suspected biliary tract pathology underwent balloon-assisted enteroscopy in a tertiary-care center. Retrospective analysis of 95 consecutive therapeutic ERCP procedures was performed to define and compare success and complication rate of DBE and SBE.

**Results:**

Male-female ratio was 28/45 with a mean age of 58 ± 2 years. 30 (32 %) procedures were performed with DBE and 65 (68 %) with SBE. Overall ERCP success rate was 73 % for DBE and 75 % for SBE (*P* = 0.831). Failure was due to inability to reach or cannulate the intact papilla or bilioenteric anastomosis. Success rate was significantly higher when performed at the bilioenteric anastomosis (80 % success in 56 procedures) or at the intact papilla in short-limb Roux-en-Y (80 % in 15 procedures) as compared to the intact papilla in long-limb (58 % in 24 procedures; *P* = 0.040). Adverse event rates were 10 % (DBE) and 8 % (SBE) (*P* = 0.707) and mostly dealt with conservatively.

**Conclusions:**

ERCP after Roux-en-Y altered small bowel anatomy is feasible and safe using both DBE and SBE. Both techniques are equally competent with high success rates and acceptable adverse events rates. ERCP at the level of the intact papilla in long limb Roux-en-Y is less successful as compared to short-limb or bilioenteric anastomosis.

## Background

Roux-en-Y enteroenteric anastomosis of the small bowel is a widely used surgical technique to drain the biliopancreatic system via an afferent jejunal limb, commonly used in gastrectomy procedures, bariatric and biliopancreatic surgery [1]. However, this type of surgery may predispose to postoperative biliary adverse events like cholangitis and common bile duct stones [2–4]. The Roux-en-Y small bowel reconstruction excludes the afferent limb and the biliary tree from conventional endoscopic access. Therefore, biliary adverse events often need percutaneous or even surgical intervention since endoscopic retrograde cholangiopancreatography (ERCP) using a conventional side-viewing duodenoscope is often not possible [5].

With respect to ERCP, Roux-en-Y reconstructions are divided in short-limb (<50 cm) and long-limb (≥100 cm) Roux-en-Y on the one hand, and bilioenteric/pancreatoenteric anastomosis versus intact Vater’s papilla on the other hand. Short-limb Roux-en-Y is used for bilioenteric/pancreatoenteric anastomosis and for (partial) gastrectomy with intact papilla, whereas long-limb Roux-en-Y in combination with intact papilla is used in several bariatric malabsorption surgical procedures [6].

Device-assisted enteroscopy, encompassing double-balloon (DBE), single-balloon (SBE) and spiral enteroscopy (SE) allows deep and even complete intubation of the small bowel [7, 8]. During DBE and SBE, a push-pull technique with a balloon-fitted overtube with (DBE) or without (SBE) a second balloon at the tip of the enteroscope is used to traverse the small bowel, whereas during SE a clockwise rotating overtube is employed. Fujinon DBE is commercially available since 2003 and Olympus SBE since 2007. Numerous studies have been performed in search of the best method of device-assisted enteroscopy [9–12]. In addition, device-assisted enteroscopy also allows intubation of the Roux-en-Y afferent limb to perform ERCP, although success rates vary [6, 13–17].

However, studies comparing different enteroscopy methods to perform ERCP after Roux-en-Y are scarce and often hampered by a small sample size. Current available literature suggests equal success rates between various types of assisted-enteroscopy [18–21].

The aim of this study is to allow a solid comparison of the feasibility of DBE and SBE to perform ERCP in patients with Roux-en-Y altered small bowel anatomy.

## Methods

From June 2006 to November 2012 a total of 95 balloon-assisted ERCP procedures for suspected biliary pathology were performed in 73 patients with Roux-en-Y altered small bowel anatomy. All procedures were performed in a high-volume tertiary-care liver transplant center. DBE was introduced in our Endoscopy unit in 2005 and SBE in 2008. Procedures were performed by one endoscopist (TGM), experienced both in ERCP and balloon-enteroscopy, with the EN-450 T5 DBE (Fujinon, Osaka, Japan) or the SIF Q180 SBE (Olympus Medical Systems, Tokyo, Japan) according to availability and preference without specific randomisation. Although there was no specific randomisation, in retrospect, SBE was chosen more often than DBE. This can be explained by the shorter preparation time (no need to mount a balloon on the tip of the SBE enteroscope) and the faster procedure steps (one balloon less to in- and deflate), as has previously been shown [22]. Both enteroscopes have a working length of 200 cm and a 2.8 mm diameter working channel allowing the use of all conventional accessory tools provided a length of at least 230 cm. Room air was used for insufflation. All procedures were performed in supine position under general anaesthesia with endotracheal intubation and under fluoroscopic control. Cannulation catheter (PR-Y0001), guidewire (G-Y0001), sphincterotome (KD-Y0005), needle knife (KD-Y0001), extraction balloon (B-Y0003), retrieval basket (FG-Y0003) and stent pusher (MAJ-Y0025-1) were Olympus prototypes (Olympus Medical Systems Corp., Japan). Conventional esophageal dilation balloons (CRE Microvasive, Boston Scientific, Ireland) were used to perform sphincteroplasty and plastic stents were conventional 7 Fr biliary stents (Cook Medical, Ireland).

Success of the ERCP procedure was defined as the accomplishment of endoscopic therapy when indicated. Diagnostic cholangiography without therapeutic intervention was not considered successful, unless diagnostic ERCP was the sole purpose of the procedure (*n* = 4). Adverse events were defined as severe adverse events leading to prolonged hospital stay with additional treatment. The learning curve was established by presenting progressive success rates (in %) per calendar year throughout the study period.

The Antwerp University Hospital Ethical Committee approved the balloon-assisted ERCP procedures and all patients consented to undergo the procedure.

Descriptive statistics were used for the patients’ characteristics, ASA status, therapeutic interventions with success and adverse event rates and were calculated using GraphPad Prism 7 (GraphPad Software Inc., USA). Comparative statistics (unpaired Student’s t-test, Mann-Whitney *U* test and Fisher’s Exact test) were used whenever appropriate to compare DBE and SBE results using GraphPad Prism 7 (GraphPad Software Inc., USA). *P* value of less than 0.05 was considered to be statistically significant.

## Results

From June 2006 onwards a total of 95 consecutive ERCP procedures were performed because of suspected biliary pathology in 73 patients with Roux-en-Y small bowel reconstruction. The male/female ratio was 28/45 (38 vs 62 %) with a mean age of 58 ± 2 years (range 19–89). Differences between patient characteristics in the DBE and the SBE group are shown in Table 1.

**Table 1:** Comparison of patient characteristics between DBE and SBE groups

	DBE	SBE	
Number of procedures:	*n* = 30	*n* = 65	*P*-value
Male/female			
Number of patients*:	10/10	20/36	0.295
Mean age years ± sem (range)	60 ± 3 (30–76)	56 ± 2 (19–89)	0.282
ASA status (median) (range)	2 (2–4)	2 (2–4)	0.720
Type of Roux-en-Y (n)			
SA	24/30	32/65	0.007
SP	2/30	13/65	0.133
LP	4/30	20/65	0.080

* Three patients underwent ERCP using both DBE and SBE, explaining the difference in total number of patients (*n* = 73) and the number of patients in the DBE (*n* = 20) and the SBE (*n* = 56) group. Statistical analysis was performed using Fisher’s Exact test (relative distribution), Student’s t test (parametric data) and Mann-Whitney *U* test (nonparametric data)

Table 2 shows the number of ERCP procedures performed in the different types of Roux-en-Y reconstructions. They are divided into 3 groups. SA: short limb (<50 cm) with bilioenteric anastomosis, as in Whipple pancreaticoduodenectomy and (liver transplantation) biliary diversion. SP: short limb (<50 cm) with intact papilla, as in total gastrectomy. LP: long limb (>100 cm) with intact papilla, as in Scopinaro biliopancreatic diversion and gastric bypass. ERCP indication was always biliary pathology, based on disturbed liver function tests and/or radiological abnormalities. None of the patients underwent endoscopic ultrasound because of the altered postoperative anatomy. All ERCP interventions performed are listed in Table 3, according to the type of Roux-en-Y reconstruction. In general, conventional biliary ERCP interventions are possible. However, due to the length and the calibre of the working channel, only plastic 7 Fr stents can be used. There is currently no metallic stent deployment system available compatible with either DBE or SBE.

**Table 2:** Types of Roux-en-Y reconstructions

	Number of procedures
1. **S**hort-limb Roux-en-Y (<50 cm) with bilioenteric **a**nastomosis (**SA**)	
Whipple pancreaticoduodenectomy	*n* = 11 (12 %)
Biliary diversion	*n* = 35 (36 %)
Liver transplantation with biliary diversion	*n* = 10 (11 %)
2. **S**hort-limb Roux-en-Y (<50 cm) with intact **p**apilla (**SP**)	
Total gastrectomy	*n* = 15 (16 %)
3. **L**ong-limb Roux-en-Y (>100 cm) with intact **p**apilla (**LP**)	
Scopinaro biliopancreatic diversion	*n* = 2 (2 %)
Gastric bypass	*n* = 22 (23 %)

**Table 3:** ERCP procedures performed according to type of Roux-en-Y reconstruction

ERCP procedure	SA	SP	LP
Cholangiogram	+	+	+
Biliary anastomosis balloon dilatation (to 12 mm)	+	-	-
Sphincterotomy	-	+	+
Precut sphincterotomy	-	+	-
Sphincteroplasty (to 12 mm)	-	+	+
Biliary stone removal	+	+	+
Biliary plastic stent (7 Fr) placement/removal	+	+	+
Removal of surgical sutures at biliary anastomosis	+	-	-
Treatment of biliary metallic stent ingrowth	-	+	-

**Fig. 1:**
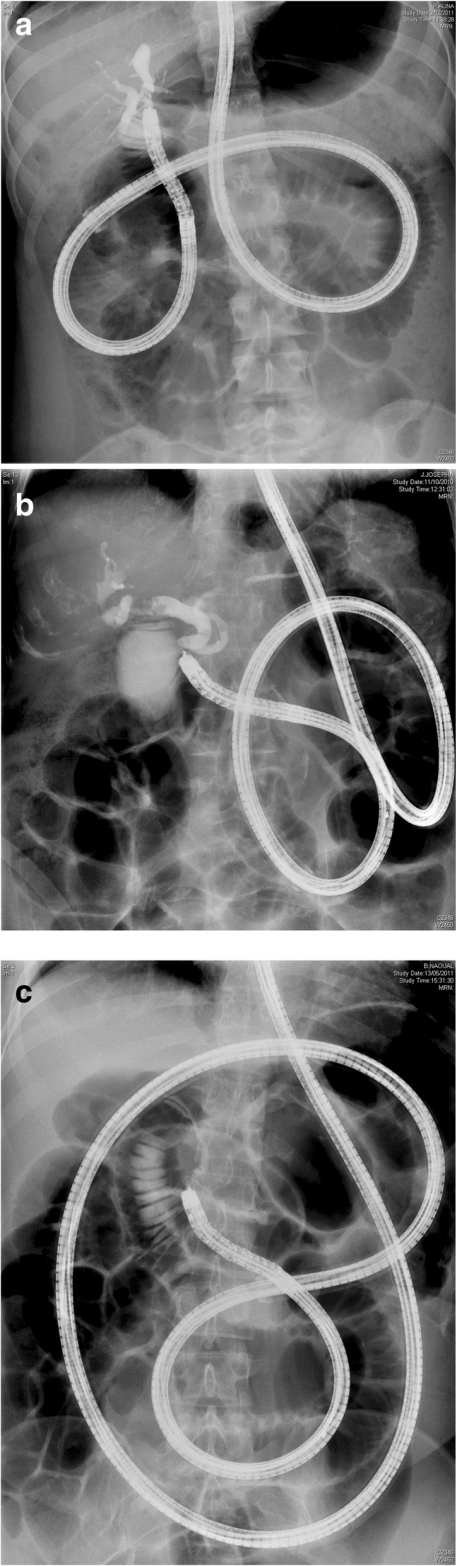
**a** Fluoroscopic image of the position of the enteroscope to obtain a cholangiogram in a 20-year old female patient with a history of a complicated cholecystectomy resulting in a Roux-en-Y bilioenteric anastomosis (SA). **b** Fluoroscopic image of the position of the enteroscope to obtain a cholangiogram in a 81-year old female patient with a history of total gastrectomy with Roux-en-Y reconstruction (SP). **c** Fluoroscopic image of the position of the enteroscope to obtain a cholangiogram in a 42-year old female patient with a history of gastric bypass bariatric surgery with Roux-en-Y reconstruction (LP)

Figure 1a–c shows fluoroscopic images of the position of the enteroscope to obtain a cholangiogram in patients with Roux-en-Y altered small bowel anatomy (SA vs SP vs LP). Cholangiography was always attempted using the PR-Y0001 cannulation catheter, and if successful cannulation was achieved, the G-Y0001 long guidewire was introduced deeply into the intrahepatic bile ducts in order not to lose its position while changing the cannulation catheter for a dilation balloon in case of bilioenteric anastomosis or the KD-Y0005 sphincterotome in case of intact papilla. Precut sphincterotomy was performed using the KD-Y0001 needle knife in case of unsuccessful cannulation of the intact papilla. Since the forward-viewing enteroscope’s position is not always stable and since it lacks a forceps elevator modality, usually only a minor sphincterotomy was performed, followed by a sphincteroplasty balloon-dilation up to 12 mm using the CRE esophageal dilation balloon. Stone extraction was performed using the B-Y0003 extraction balloon or the FG-Y0003 retrieval basket. When indicated, conventional 7 Fr plastic biliary stents where pushed in place over the guidewire using the MAJ-Y0025-1 pusher. Conventional 10 Fr biliary stents cannot be used due to the diameter mismatch between the stent (>3 mm) and the enteroscopes’ working channel diameter (2.8 mm). Finally, we treated 3 patients with recurring cholangitis after Roux-en-Y bilioenteric anastomosis due to clotted debris entrapped in unresolvable sutures used for the bilioenteric anastomosis. Sutures were removed using an endoscopic scissor.

**Fig. 2:**
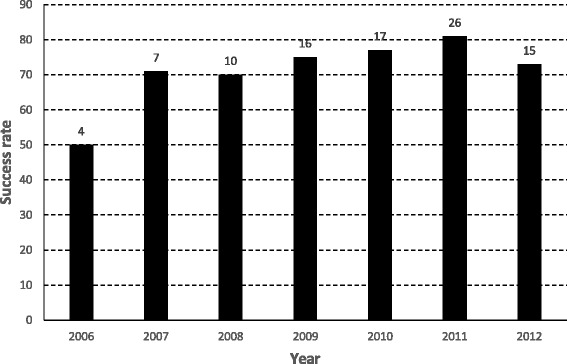
DBE and SBE learning curve of Roux-en-Y ERCP

Thirty (32 %) ERCP procedures were performed with the Fujinon DBE and 65 (68 %) with the Olympus SBE. Overall ERCP success rate was 74 % (70 out of 95 procedures) with 73 % in DBE and 75 % in SBE procedures (*P* = 0.806). Overall adverse events rate was 8 % (8 out of 95 procedures) with 10 % in DBE and 8 % in SBE procedures (*P* = 0.704). No significant differences of success and adverse events rates between DBE and SBE were encountered. However, throughout the years of the study period, therapeutic success rate increased from 50 % in the first study year (2006) to over 70 % since 2007 and on. DBE was introduced before SBE in our Endoscopy Unit, and there was a clear increase in ERCP procedures in Roux-en-Y patients from 2009 onwards, as shown in the learning curve success rate of the 6 years of balloon-assisted ERCP in patients with Roux-en-Y small bowel altered anatomy (Fig. 2). In addition, success rate was significantly higher when ERCP was performed in patients with short-limb Roux-en-Y, both at the biliary anastomosis in SA (80 % success in 56 procedures) or at the intact papilla in SP (80 % in 15 procedures) as compared to long-limb Roux-en-Y with intact papilla LP (58 % in 24 procedures; *P* = 0.040).

Serious adverse events occurred in 8 procedures, 3/30 with DBE (10 %) and 5/65 with SBE (8 %) (*P* = 0.704). There were 3 cases of retroperitoneal free air after sphicterotomy and sphincteroplasty which were all treated conservatively by means of intravenous antibiotics and in 1 case intravenous somatostatin. There were 2 cases of post-ERCP cholangitis which were treated with intravenous antibiotics and 2 with post-ERCP pancreatitis with normal outcome after conservative treatment. Finally, 1 case of liver capsula dehiscence because of guide wire perforation resulted in surinfected subcapsular liver hematoma and needed surgical drainage.

## Discussion

Roux-en-Y small bowel reconstruction is a frequently used surgical technique in hepato-bilio-pancreatic surgery and gastric and bariatric surgery [1]. However, it may predispose to the development of biliary adverse events like cholangitis due to bilioenteric anastomotic stenosis or bile duct stones [2–4]. Due to the Roux-en-Y reconstruction, these adverse events cannot be treated endoscopically with a conventional side-viewing duodenoscope used for ERCP. With the development of device-assisted enteroscopy (DBE, SBE and SE), the biliary tract of patients with Roux-en-Y altered anatomy has come into endoscopic reach [6]. And its use to perform ERCP in patients with Roux-en-Y altered anatomy has been shown using DBE, SBE, short DBE and short SBE and SE [13–18, 23]. However data on direct comparison between the different device-assisted enteroscopy methods are scarce. We previously showed that 4 patients with Roux-en-Y bilioenteric anastomosis stenosis were repeatedly treated with endoscopic balloon dilation using both DBE and SBE with similar success [18]. Others have shown similar ERCP success rates in Roux-en-Y patients using SBE and SE [19]. A multicentre retrospective study also showed comparable results using DBE, SBE and SE with an overall ERCP success rate of 63 % both in long-limb and in short-limb Roux-en-Y patients [20].

We achieved a higher ERCP success rate in patients with short-limb Roux-en-Y (80 %) and comparable ERCP success rate in patients with long-limb Roux-en-Y (58 %), which might reflect the difference between a multicenter and a single center retrospective study. Finally, a Japanese study showed comparable therapeutic success using SBE and short SBE [21]. In the current manuscript we present data on a large cohort of 95 ERCP procedures in 73 Roux-en-Y patients performed by a single endoscopist using both DBE and SBE. Retrospective analysis showed comparable therapeutic success rates for DBE (73 %) and SBE (75 %) and comparable adverse events rates (10 vs 8 %) without procedure-related mortality. As suggested before, the present study confirms that similar ERCP success rates are obtained in Roux-en-Y patients independent of the balloon-assisted enteroscopy method. Based on the positive learning curve, adequate training to become familiar with Roux-en-Y ERCP seems predominant in determining success rate irrespective of the enteroscopy method itself. We showed that overall therapeutic success rates increased throughout the study period, from 50 % during the first year to over 70 % thereafter. This illustrates the challenging nature of each individual Roux-en-Y ERCP procedure even in well-trained hands.

In our series, only the length of the Roux-en-Y limbs seemed to be a determining factor of ERCP success and not the presence of intact papilla or bilioenteric anastomosis. Although it was suggested before that an intact papilla is more difficult to cannulate as compared to a bilioenteric anastomosis, this was not the case in our large cohort [17]. Longer Roux-en-Y limbs decreased therapeutic success rates by limiting the access to the papilla. However, others have shown that even with the recently developed shorter versions (152 cm) of both DBE and SBE, ERCP was feasible in patients with altered intestinal anatomy, even in patients with long-limb Roux-en-Y [21, 23–25]. It seems however acceptable that longer Roux-en-Y limbs may render reaching the intact papilla more difficult, with a negative impact on therapeutic success rate.

Despite a narrow working channel, with a diameter of 2.8 mm, currently available balloon-assisted enteroscopes allow all conventional ERCP interventions apart from placement of through-the-scope metal stents and conventional 10 Fr plastic stents: (precut) sphincterotomy with sphincteroplasty or balloon dilation of bilioenteric anastomosis stenosis, stone extraction and plastic stent placement and removal. However, dedicated accessory material is mandatory since conventional ERCP accessories are not long enough. Catheters should have a length of at least 230 cm and guidewires should be at least double that length in order to allow correct exchange of the catheters. Further development of these accessory tools is mandatory to enable improvement of Roux-en-Y ERCP. The recent development of a short-type SBE with a 3.2 mm working channel will allow the use of more conventional ERCP tools in patients with short limb Roux-en-Y reconstruction [26].

Perforation, either retroperitoneal or anastomotic (bilioenteric and enteroenteric), seems the most common serious adverse event of Roux-en-Y ERCP, also in our series [17]. All cases were treated conservatively without the need for surgical intervention. One adverse event of a major subcapsular hepatic hematoma deserves further attention. This specific adverse event probably resulted from the combination of a ‘closed loop’ phenomenon and deep introduction of the guidewire into the intrahepatic biliary tree. The patient was an 81-year old male with a Roux-en-Y total gastrectomy (SP) referred for ERCP because of common bile duct stones. Sphincterotomy and sphincteroplasty followed by stone extraction with the extraction balloon was successful. However, during the procedure, the afferent Roux-en-Y loop was closed by the inflated balloon of the SBE overtube and air was insufflated through the enteroscope leading to increased intraluminal and intraductal pressure. The guidewire has to be positioned deeply into the intrahepatic ducts in order not to lose its position while changing catheters over the guidewire without forceps elevator modality. The guidewire may have traumatised the peripheral liver parenchyma without rupture of the capsula. The increased intraluminal and intraductal pressure of the closed loop following the use of air insufflation has led to a capsule dehiscence and bleeding. Intermittent desufflation of the enteroscope’s balloon and overtube’s balloon during the procedure to release intraluminal pressure is advisable. In addition, the use of CO_2_ insufflation may decrease this type of barotraumas [17].

The advantage of the current study is that all DBE and SBE procedures were performed by one single endoscopist, avoiding bias in the comparison of both techniques. However, as shown by the positive learning curve, earlier procedures were less successful. Since SBE became only available in our endoscopy unit in 2008, the first 11 ERCP procedures were all performed using DBE. Since 2008, DBE and SBE were randomly used according to availability and endoscopist’s preference and showed no specific difference in therapeutic success rate. Although there was no specific randomisation, in retrospect, SBE was chosen more often than DBE. This was explained by the shorter preparation time (no need to mount a balloon on the tip of the SBE enteroscope) and the faster procedure steps (one balloon less to in- and deflate), as has previously been shown [22].

As this is a retrospective analysis of endoscopic data, the study contains several limitations potentially leading to confounding factors in the interpretation of the results. DBE was available from 2005 and SBE from 2008, meaning that the ERCP learning curve was largely dependent on DBE, which may have impaired the DBE success rate. In contrast to this, there was an imbalance in the relative distribution of the 3 types of Roux-en-Y surgery (SA, SP and LP) between DBE and SBE, with SA being relatively better represented in the DBE group as compared to the SBE group (Table 1). However, ERCP in a patient with a SA type of Roux-en-Y reconstruction showed more successful than with LP type Roux-en-Y, favouring the DBE success rate. This imbalance also reflects the fact that in the first years of experience (when only DBE was available), less complicated cases (SA) were referred for ERCP. A final limitation is the lack of CO_2_-insufflation during the study period. It is advised to limit air-insufflation and to use CO_2_-insufflation for complex therapeutic endoscopic procedures like ERCP [27].

## Conclusions

DBE and SBE have high and comparable therapeutic success rates and acceptable adverse events rates, irrespective of the type of balloon-assisted enteroscopy method to perform ERCP with biliary access in patients with Roux-en-Y altered anatomy. The most important determining factor of therapeutic success seems the length of the Roux-en-Y limb. Further development of the equipment (both endoscopes and catheters) is mandatory to improve the success of Roux-en-Y ERCP and to make it the first method of choice to deal with biliary pathology in patients with Roux-en-Y altered small bowel anatomy.

## Abbreviations

ASA: American Society of Anesthesiology
DBE: Double-balloon enteroscope
ERCP: Endoscopic retrograde cholangiopancreatogram
LP: Long-limb Roux-en-Y with intact papilla
SA: Short-limb Roux-en-Y with bilioenteric anastomosis
SBE: Single-balloon enteroscope
SE: Spiral enteroscopy
SP: Short-limb Roux-en-Y with intact papilla
